# Report of the Sanitary Committee of New Orleans on the Epidemic Yellow Fever of 1853

**Published:** 1855-02

**Authors:** 


ART. V.—*Report of the Sanitary Commission of New Orleans on the Epidemic Yellow Fever of* 1853; *published by Authority of the City Council of New Orleans.*

——Quod sol atque imbres dederant, quod
Terra crearât sponte sua.

LUCRET. LIB. V.

*Report of the Select Committee of the Senate of the United States on the Sickness and Mortality on board Emigrant Ships, Aug.* 2, 1854. (Hon. Hamilton Fish, of N. Y., Chairman.)

We have coupled together these two reports for obvious reasons. Both are official in their character. Both are emanations from that humane principle in political economy which makes the health of the citizen one of the cares of the government. Both appeal at once to patriotism and humanity for the amelioration of that unfortunate class who are our hewers of wood and drawers of water, who build our railroads and dig our canals, and whose own intelligence is not sufficient to protect them from those evils which follow ignorance as sure as punishment follows crime. And both are honest, intelligent, noble vindications of that great principle of modern medicine which asserts that a large class of diseases (all of the "zymotics") are within the control of human agencies, preventable by human means, and developed only as the just penalty of human sins against the laws of health.

Nowhere does the dignity of medicine so vindicate itself, nowhere does it so assume its rightful position as the guardian angel of humanity, as when it points to the hidden sources of disease and teaches how to shun them.

Guyot, in "Earth and Man," speaks of life as an interchange of relations and extends the meaning of the word to the inorganic forms of matter. The principle which combines oxygen and hydrogen to make water, is an effort of life. As the relations of chemical combinations grow more complex we have a more intricate exchange of relations, until finally, it develops itself with the powers of locomotion, generation, and secretion, and is manifested as that sentient existence which is known as animal life. The less complex these relations, the less liable to disturbance are they. But as in human life, in the transition from savage to civilized existence, the exchange of relations becomes more complex, the simple habits of barbarian life passing into the arts, the architecture, the commerce of civilization, this very complicity of relations brings with it the penalties of easy disturbance, of variety in disease, and of new duties for the scientific and benevolent in ameliorating the evils consequent upon an artificial manner of existence.

The shepherd, the husbandman, and the hunter, the three simple forms of primitive life, enlarge into the three hundred and thirty occupations and trades enumerated by the last British census. Man leaves the fields and aggregates in cities, the simple relation of the isolated family is merged in those associated efforts which send hundreds together across oceans and continents, and the result is found in the generation of a thousand offspring of Pandora to be strangled by the hand of science.

The large quotations from Dr. Barton's Report, given in our last issue, might pass as a sufficient notice so far as its practical views are concerned. These are not claimed to be original. Dr. Barton urges upon the authorities the old means of prevention. With him, sewerage, paving, cleanliness, are the Trinity whose unit is health, so far as municipal means can act. But here the old problem occurs. New Orleans is always unpaved, undrained, and uncleanly. Why, then, shall it not always have yellow fever?

The triad of causes enumerated are to use Dr. Barton's forcible comparison "but one blade of the shears." Another is wanting to give it efficiency, and this other is a cause inconstant, and except to the eye of science inappreciable, but nevertheless forever present where the banner of the pestilence is unfurled.

Couple these "terrene" causes with the meteorological condition of a high saturation with a high temperature and "the shears are complete."

It would be difficult for us to enumerate all the arguments brought to the support of this proposition. Briefly, the principal are these: In all the yellow fever zone wherever the meteoric condition was observed, a high dew-point was the unfailing accompaniment of the disease. With what is usually called the "caprice" of epidemics one uncleanly city suffered while its equally dirty neighbor escaped. Here, those places which were kept clean were healthy though they had a high dew-point. Those that were uncleanly suffered if they had a high dew-point.

And wherever a high dew-point and uncleanliness were associated the epidemic prevailed in a direct ratio to the intensity of these two conditions.

These are the results in places where the meteoric condition was known by actual observation. In other places it was arrived at by approximation, judging from the frequency of the showers, the presence of mould, and the decay or imperfect nutrition of the fruits of the earth. Fortunately these observations, which extend over the whole yellow fever zone from Philadelphia to Rio Janeiro, are supported by the most careful testimony. They extend, too, in New Orleans, through a period of thirty years, the same conditions accompanying every epidemic.

The reasonings derived from these facts, cannot be confined to the yellow fever zone. In some articles on the health of the city of Buffalo, published during the past autumn in the Buffalo Commercial Advertiser, and thence transferred to this Journal, we found cholera confining its ravages to the low lying districts and to those situations where filth and lack of draining invited it to localize. There, also, we found the allied diseases of the summer season, and when our investigation of the favorite localities of all the zymotic diseases is completed, there we expect to find the habitat of typhus, of scarlatina, of rubeola, and the other exanthemata. Again, we may refer to the cholera of 1852 in this place, where Prof. Hamilton traced so clearly the influence of the upturning of the earth, with a stagnant atmosphere, in the immediate causation of cholera. The investigations of modern meteorology have settled the whole question of miasm. There is no longer room for two opinions upon the subject.

Returning from our discursion we wish again to call attention to that which is the most important inference from both of the reports under review. Senator Fish points out the fact that typhus, cholera, and variola, are the peculiar curses of the emigrant on ship-board — that these do not exist there without a local cause, and then demonstrating what is the cause, he calls for reforms precisely analagous to those demanded by his southern confrere in hygiene. In this laical report by a gentleman whose study has been civil policy rather than natural science, we have the calm unprejudiced convictions of a logical mind, reaching the same conclusions which are more amply proved by the researches of science.

Both, then, assert distinctly the doctrine of the preventability of zymotic disease — Senator Fish in the narrower sphere allotted to him — Dr. Barton in the whole range of zymotic disease. We believe that the evidence warrants the conclusion. The additional responsibility thus thrown upon the shoulders of governments becomes a serious study for the statesman. H.

